# Regional tau PET patterns predict prospective domain-specific cognitive decline in early symptomatic Alzheimer’s disease

**DOI:** 10.1186/s13195-025-01868-7

**Published:** 2025-10-06

**Authors:** Maura Malpetti, Saima Rathore, Leonardo Iaccarino, Renaud La Joie, Giulia Tronchin, Alette M. Wessels, John R. Sims, Michael J. Pontecorvo, Sergey Shcherbinin, Gil D. Rabinovici

**Affiliations:** 1 Department of Clinical Neurosciences, Cambridge University Hospitals NHS Trust, University of Cambridge, Herchel Smith Building, Forvie Site Robinson Way, Cambridge Biomedical Campus, CB2 0SZ Cambridge, UK; 2 UK Dementia Research Institute at University of Cambridge, Cambridge, CB2 0XY UK; 3 Eli Lilly and Company, Indianapolis, USA; 4 Memory and Aging Center, Department of Neurology, Department of Neurology, Weill Institute for Neurosciences, University of California, San Francisco, San Francisco, CA USA; 5 Department of Radiology and Biomedical Imaging, University of California, San Francisco, San Francisco, CA USA

**Keywords:** Tau PET, Prognosis, Cognitive domains, Early AD

## Abstract

**Background:**

Tau-PET binding patterns are heterogenous, with regional binding showing strong cross-sectional correlations with domain-specific cognitive performance and longitudinal correlations with prospective neurodegeneration. Here we evaluated whether regional patterns of baseline tau-PET predict prospective longitudinal decline in specific cognitive domains in early symptomatic Alzheimer’s disease (AD), including participants from clinical trial cohorts.

**Methods:**

731 amyloid-positive participants with a clinical diagnosis of mild cognitive impairment (MCI) or mild AD dementia underwent a flortaucipir F 18 PET (FTP-PET), structural MRI, and neuropsychological testing with the AD Assessment Scale-Cognitive Subscale (ADAS-Cog). Cognitive assessment was repeated after 9–18 or 12–24 months. Sub-scale annualized w-scores at each time point were combined as composite scores according to domains, including episodic memory, semantic memory, executive function, language and praxis. Latent growth curve models were applied to longitudinal composite scores to estimate the rate of annual decline (slope) in each participant. Standardized uptake value ratio (SUVR) images were created using cerebellar crus as the reference region. Regional and voxel-wise correlation analyses were implemented to identify baseline FTP-PET patterns and MRI grey matter volumes associated with longitudinal changes in each cognitive domain, and to evaluate whether MRI mediates the association between FTP-PET and cognitive decline.

**Results:**

Differential FTP-PET signal patterns showed significant negative associations with domain-specific annual rates of decline. Higher temporo-parietal FTP-PET SUVR was associated with faster decline in episodic memory, while higher left anterior temporal SUVR was associated with faster decline in semantic memory. FTP-PET signal in a left-dominant fronto-temporal pattern was associated with faster decline in language, while FTP-PET signal in a right-dominant fronto-parietal pattern was associated with faster decline in praxis. Executive decline showed limited spatial associations with FTP-PET. Overall, regional and voxel-wise analyses identified similar pairwise associations between FTP-PET signal and domain-specific longitudinal decline. Baseline MRI showed weaker associations with domain-specific cognitive decline than FTP-PET, and did not mediate the predictive effect of the latter.

**Conclusion:**

Differential regional tau-PET signal patterns were associated with domain-specific cognitive decline in MCI and early AD dementia. Tau-PET may be a useful precision medicine tool to support individualized predictions of cognitive decline trajectories in early symptomatic AD.

**Supplementary Information:**

The online version contains supplementary material available at 10.1186/s13195-025-01868-7.

## Background

The new era of disease modifying therapies for Alzheimer’s disease (AD) has been made possible by the utilization of biomarkers – highlighting that it is crucial to continue to validate these tools for more accurate prognosis and stratification of patients to advance disease-modifying treatments through clinical trials. Several biomarkers have emerged to quantify and track AD pathological hallmarks, including positron emission tomography (PET) radiotracers that bind selectively to amyloid-β plaques and tau neurofibrillary tangles. Whereas in vivo amyloid PET, as well as amyloid neuropathology at postmortem, correlate only weakly with cognitive impairment cross-sectionally, tau load assessed by tau-PET strongly correlates with clinical progression and the extent of neurodegeneration [1–3].

Cross-sectional studies show that tau-PET uptake topography overlaps with patterns of brain atrophy and hypometabolism as measured by magnetic resonance imaging (MRI) or FDG-PET [4, 5]. Tau-PET is also associated with domain-specific cognitive deficits [6–9] and distinct clinical variants of AD [5, 10, 11]. Longitudinally, strong associations between baseline tau-PET and cognitive changes over time have been described across the AD clinical spectrum. Tau-PET uptake in temporo-parietal regions outperforms amyloid-PET, structural MRI measures and fluid biomarkers in predicting cognitive decline on global scales [2, 12–15]. This has been replicated in several single- and multi-center studies, with different tau-PET tracers and participants at different stages of the disease course. These converging findings suggest that tau-PET is a promising prognostic tool for predicting cognitive decline.

Current disease-modifying treatment trials commonly rely on global/composite measures of cognitive and functional decline to assess the efficacy of the intervention. While being useful and easily implementable, this approach may not take into account the variety of AD-related clinical presentations, with non-amnestic syndromes and individual decline profiles in potentially different cognitive domains. Similarly, most previous evidence on the prognostic value of tau-PET has focused on global or summary metrics of tracer uptake or regional uptake in a single region (e.g., temporal meta-ROI) to predict global measures of cognitive decline or changes in the memory domain specifically. This approach captures initial tau accumulation, linking mesial temporal structures with memory performance, however neglects relevant information about differential spatial patterns that tau-PET can reveal. Identifying more granular associations between tau PET patterns and domain-specific decline has the potential to complement traditional global tau-cognition associations and support both disease staging and prognosis as well as potentially serve as potential outcome measure in clinical trials.

Here, we aim to test whether tau-PET spatial patterns are useful to *track* and *predict* differential cognitive trajectories on the AD spectrum in clinical-trial cohorts. Specifically, we evaluated whether regional patterns of baseline tau-PET predict prospective longitudinal decline in specific cognitive domains in early symptomatic AD. Our study focused on 18F flortaucipir (FTP-PET), which is to date the only tau-PET tracer to have received FDA and EMA-approval for clinical use in the U.S./EU. In a cohort of 731 patients with AD, we tested whether (i) baseline differential tau-PET patterns correlate with domain-specific cognitive performance cross-sectionally; (ii) similar tau-PET patterns at baseline predict cognitive decline in specific domains over time; (iii) structural brain changes over time mediate the prognostic value of tau PET. Establishing tau-PET as a tool for tracking in vivo disease and predicting clinical progression would improve patient prognostication and management, also potentially enriching screening and outcome evaluation in clinical trials.

## Materials and methods

### Study participants

We included participants enrolled either in observational flortaucipir clinical development studies (AV1451-A05: NCT02016560 [2]) or in interventional clinical trials (EXPEDITION-3: NCT01900665 [16], and AMARANTH: NCT02245737 [17]) conducted at Eli Lilly and Company. Seven hundreds thirty-one (*N* = 731) participants were included in our analyses, as they: (i) had a clinical diagnosis of mild cognitive impairment (MCI) due to AD, or AD with mild dementia based on NIA-AA criteria ; (ii) had a positive florbetapir PET scan (assessed via visual interpretation with adjunct quantitative information - the VisQ method [18] - evaluating cortical Aβ levels); (iii) underwent a FTP-PET PETscan at baseline; (iv) had available clinical and neuropsychological assessment data. Out of 731 participants, 221 had a diagnosis of MCI due to AD, and 510 had a diagnosis of AD with mild dementia.

For longitudinal analyses, we included all participants from the observational study (AV1451-A05; *n* = 108), and participants in placebo arms only from the two clinical trials (EXPEDITION-3 *n* = 82, and AMARANTH *n* = 31), while participants in treatment arms were excluded.

### Image acquisition and processing

In study A05, flortaucipir PET images were acquired across four 5-min frames (20 min total) beginning 80 min after IV injection of 370 MBq flortaucipir F18 as described in previous publications. In Expedition 3 and Amaranth images were acquired across six 5-min frames (30 min total) beginning 75 min after IV injection of 240 MBq flortaucipir F18. Other aspects of image acquisition and processing were comparable across the three studies and methods have been described in earlier publications [2, 19]. Briefly, all FTP-PET scans were motion- and time-corrected, followed by summing into a single scan. After a series of pre-processing steps [2], the summed FTP-PET scans were transformed to Montreal Neurologic Institute (MNI) atlas space. Automated anatomical labelling (AAL) atlas was used to partition the spatially-normalized FTP-PET images into 92 anatomical regions. Voxel-wise maps and regional SUVR values were created using subject-specific cerebellum crus as reference region. Cortical surface maps were created for baseline and follow-up FTP-PET scans of each participant. Cortical reconstruction program provided by FreeSurfer (version 6.0.0w) was applied on the baseline T1-weighted MRI scans to segregate white matter and pial surfaces and to calculate subject-specific subcortical segmentations. The cortical reconstructions with macroscopically failed segmentations and/or surface errors were removed from further analysis after manual inspection. The motion- and time-corrected baseline and follow-up FTP-PET scans were registered to the bias-corrected T1-weighted MRI scans generated by FreeSurfer using Linear Image Registration Tool with 6 degrees-of-freedom. The FTP-PET uptake halfway between the pial and white surfaces was projected onto FreeSurfer’s average template and smoothed by a white surface Gaussian filter with a full width at half maximum (FWHM) of 5 mm. The smoothed surface maps were then normalized by each subject’s cerebellum crus reference value to produce FTP-PET reflecting standardized uptake volume ratio (SUVR) surface. Unlike conventional approaches that rely on atlas-based inferior cerebellar gray matter or whole cerebellum regions, our method uses a subject-specific cerebellar crus reference region derived from each individual’s T1-weighted MRI. The inferior cerebellar gray matter is located at the bottom of the PET field of view, thus attenuation correction and spatial normalization can be more prone to error. Our approach allowed for an accurate placement of the reference region, especially in cases with severe atrophy, while maintaining consistency across participants.

### Cognitive assessment and domains

At baseline, all participants underwent the ADAS-Cog cognitive assessment, which evaluates episodic and semantic memory, language, executive and praxis functions (higher scores indicate worse performance in each of these domains). The same cognitive assessment was repeated after 9 and 18 months in A05 and EXPEDITION-3 trials, and after 1 year and 2 years in AMARANTH. Sub-scores for episodic memory were calculated including the recall, recognition, orientation, and remembering instructions items from the ADAS-Cog cognitive scale. Semantic memory was measured using the naming objects and fingers ADAS-Cog sub-scores, while the language sub-score included scores in difficulty in finding words and following commands. Executive functions were evaluated with the number of cancellations from ADAS-Cog13, and available for the AMARANTH and EXPEDITION-3 studies but not in the A05 study. Finally, the ideational and constructional praxis subscores were used to assess the praxis domain.

To obtain comparable scores across domains, we calculated patient-specific W-scores (age-corrected z-scores) for each ADAS-Cog sub-score against a group of 68 cognitively unimpaired, amyloid-negative controls (recruited as part of the A05 study; female: 46.3%; apoe4-carriers: 20%; percentage white: 75%; mean (SD) age: 58.94 (18.68); mean years of education: 15.83; all with a Mini-Mental State Examination (MMSE) > 28, average MMSE (SD): 29.50 (0.50); average ADAS-Cog11 (SD): 5.34 (3.28)). Notably, this group included 15 young controls with a mean age of 29.0. Next, patient-specific W-scores of single ADAS-Cog sub-tests were averaged within each cognitive domain. None of the patients included had missing cognitive assessment data. The executive domain was evaluated only in the AMARANTH and EXPEDITION-3 studies, as the respective test of number of cancellations from ADAS-Cog13 was not available in A05 study. Moreover, since the normal controls were not available in AMARANTH and EXPEDITION-3 for calculating w-scores, raw scores were used for the executive domain.

### Statistical analysis

First, ROI-based Pearson correlation analyses were performed to identify the cross-sectional associations between left and right regional tau-PET SUVR values and domain-specific cognitive impairment at baseline. Second, we applied latent growth curve models (LGCM) to longitudinal domain-specific scores to estimate the rate of annual decline (slope) for each participant. Worse cognitive performance is reflected in higher scores on the ADAS test over time. Domain-specific scores at follow-up were annualized to the nearest whole year for A05 and EXPEDITION3 studies, using the absolute difference in scores between the baseline and the following visits, divided by the time interval in days between tests and multiplied by 365 (9 months converted to 1 year) and 730 (18 months converted to 2 years). This allowed us to run the models across all cohorts using equidistant and comparable time intervals between visits, and to calculate slopes that are interpretable as ‘annual rate of change’ in each cognitive domain. LGCM were fitted on longitudinal annualized domain-specific scores across all patients, and linear slopes for domain-specific scores were estimated, and used in further analyses. LGCM was implemented in Lavaan software using full information maximum likelihood estimation with robust standard errors for missingness and non-normality. We considered three indices of good model fit [20]: (1) the root-mean-square error of approximation (RMSEA, acceptable fit: < 0.08, good fit: < 0.05), (2) the comparative fit index (CFI, acceptable fit: 0.95–0.97, good fit: >0.97), and (3) the standardized root mean-square residual (SRMR, acceptable fit: 0.05–0.10, good fit: < 0.05). From the model fitting, variables “intercept” and “slope” were created extracting the individual estimated values for each subject in the model. Baseline tau-PET SUVR regional values were included in region-specific correlation analyses with the annual rate of domain-specific decline (slope). Associations between tau-PET and domain-specific cognitive impairments, cross-sectionally and longitudinally, were also tested with voxel-wise regression analyses. Both ROI-based and voxel-wise analyses were performed across all patients, and within each diagnostic group (MCI, dementia). Results were considered statistically significant with a *p* < 0.05 with Family Wise Error (FWE) correction, and cluster extent k = 100 was used for voxel-wise regression analyses. Covariates were not included as the approach already accounted for age (calculating w-scores) and baseline clinical severity (LGCM account for intercept and slope covariance). Analyses adjusting for sex as covariate led to the same results, thus we did not include this in the final analyses.

Next, we tested whether the relationship between baseline tau-PET and longitudinal domain-specific cognitive decline was mediated by atrophy in the same brain regions. First, we tested for associations between gray-matter regional volumes and cognitive change slope. Second, we undertook mediation analyses within specific brain regions where cognitive impairment showed associations with both tau-PET SUVR and grey matter volume. Our analysis was restricted to those regions where imaging-cognition correlations with both imaging modalities, thus ensuring an impartial assessment without favoring one modality over the other. Utilizing the ‘mediation’ R package, we computed the average direct effect (ADE) and the average causal mediation effect (ACME). These effects were estimated as population averages through non-parametric bootstrapping comprising 5000 simulations, with a significance level set at *p* < 0.05.

## Results

Table 1 shows demographics and summaries of patient w-scores across various cognitive domains of participants included in cross-sectional analyses. Nearly half of the sample (374/731) were female, and 66% of the sample (478/720; data for 11 patients was not available) were APOE-ε4 carriers. One-sample t-tests reveal significant impairment in all cognitive domains, suggesting poorer performance compared to controls. Specifically, these differences were observed in episodic memory [t = 24.56, *p* < 0.0001], semantic memory [t = 12.24, *p* < 0.001], language [t = 18.16, *p* < 0.001], executive [t = 27.40, *p* < 0.001], and praxis [t = 15.96, *p* < 0.001]. Domain-specific cognitive performance did not differ significantly across study cohorts at the statistical threshold of *p* < 0.05. Table 2 shows the demographics and clinical/cognitive assessment summaries for participants (*n* = 221; 65% with mild AD dementia, 35% with MCI due to AD) included in the longitudinal analyses. The average age of the cohort was 74 years; 51% were male, 64% were APOE-ε4 carriers, and the average follow-up time was 19 months.

**Table 1:** Clinical, demographics, and genetic characteristics of the 731 participants included in cross-sectional analysis

Clinical and Genetic Characteristics	Complete Dataset (*n* = 731)	A05 (*n* = 173)	AMARANTH (*n* = 365)	EXPEDITION-3 (*n* = 193)	
Age in years, mean (SD) [min-max]	73.0 (8.0) [MCI: 50–97, AD: 54–95]	74.3 (9.3) [MCI: 50–97, AD: 54–95]	71.7 (7.5) [MCI:55–85, AD:55–85]	73.9 (8.0) [AD: 55–90]	
Sex: male/female, n	357/374	90/83	178/187	89/104	
APOE-ε4: non-carriers/carriers, n	242/478 (11*)	66/101 (6*)	121/243 (1*)	55/134 (4*)	
Race					
Asian, n	56	1	41	14	
Black/African American, n	11	2	7	2	
White, n	640 (24*)	169 (1*)	314 (3*)	157 (20*)	
Ethnicity					
Hispanic or Latino, n	22	4	14	4	
Not Hispanic or Latino, n	597	91	337	169	
Clinical Diagnosis					
AD with mild dementia, n (%)	510 (69.76)	76 (43.93)	241 (66.02)	193 (100.00)	
MCI due to AD, n (%)	221 (30.23)	97 (56.06)	124 (33.97)	0 (0.00)	
Cognitive composite scores at baseline					
Episodic memory (w-score), mean (SD)	1.85 (1.92)	1.64 (1.86)	1.93 (2.02)	1.91 (1.79)	
Semantic memory (w-score), mean (SD)	0.78 (1.87)	0.95 (2.25)	0.59 (1.63)	0.98 (1.92)	
Executive function (0–5 score), mean (SD)	2.58 (1.23)	---	2.56 (1.24)	2.60 (1.21)	
Language (w-score), mean (SD)	2.92 (3.91)	2.61 (3.94)	2.84 (3.80)	3.34 (4.08)	
Praxis (w-score), mean (SD)	1.18 (2.00)	0.96 (1.91)	1.29 (2.11)	1.18 (1.86)	

Abbreviations: AD, Alzheimer’s disease; APOE, apolipoprotein E; MCI, mild cognitive impairment; n, number of participants in the analysis; SD, standard deviation. Higher w-scores correspond to worse cognitive performance. The * indicates missing information

**Table 2:** Clinical, demographics, and genetic characteristics of the 221 participants included in prognostic analysis with longitudinal cognitive assessments

Clinical and Genetic Characteristics	Complete Dataset (*n* = 221)	A05 (*n* = 108)	AMARANTH (*n* = 31)	EXPEDITION-3 (*n* = 82)	
Age in years, mean (SD) [min-max]	74.47 (8.58)	75.35 (9.03)	72.29 (7.29)	74.14 (8.18)	
Sex: male/female, n	113/108	61/47	16/15	36/46	
APOE-ε4: non-carriers/carriers, n	76/137	42/62	12/19	22/56	
Race					
Asian, n	8	0	4	4	
Black/African American, n	5	2	1	2	
White, n	199	105	26	68	
Ethnicity					
Hispanic or Latino, n	7	2	3	2	
Not Hispanic or Latino, n	156	58	26	72	
Clinical Diagnosis					
AD with mild dementia, n (%)	144 (65.16)	43 (39.81)	19 (61.29)	82 (100)	
MCI due to AD, n (%)	77 (34.84)	65 (60.19)	12 (38.71)	0 (0.00)	
Cognitive composite estimated slopes					
Episodic memory (w-score), mean (SD)	0.06 (0.60)	0.11 (0.59)	-0.04 (0.43)	0.03 (0.67)	
Semantic memory (w-score), mean (SD)	0.02 (0.24)	0.02 (0.23)	0.01 (0.17)	0.02 (0.27)	
Executive function (0–5 score), mean (SD)	0.03 (0.46)	--	0.03 (0.61)	0.03 (0.40)	
Language (w-score), mean (SD)	0.04 (0.71)	0.02 (0.62)	0.10 (0.68)	0.05 (0.84)	
Praxis (w-score), mean (SD)	0.04 (0.72)	0.04 (0.64)	0.14 (0.80)	0.00 (0.79)	

Abbreviations: AD, Alzheimer’s disease; APOE, apolipoprotein E; MCI, mild cognitive impairment; n, number of participants in the analysis; SD, standard deviation. Higher w-scores correspond to worse cognitive performance. The -- indicates missing of executive domain in A05 study

### Associations between domain-specific cognitive performance and tau-PET imaging at baseline

**Fig. 1:**
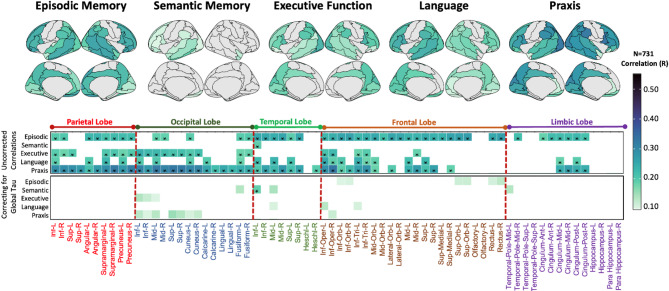
Correlations between regional tau-PET SUVR values and domain-specific cognitive impairment at baseline. The surface maps display all the regional correlations assessed, while the bottom heatmap highlights only those correlations which have *p* < 0.05. The * indicates the correlations which survived stringent threshold of pFWE < 0.05. The x-axis of heatmap groups AAL brain regions by lobes, and the y-axis represents cognitive domains

Results from regional correlations between domain-specific cognitive performance and tau-PET SUVR are reported in Fig. 1. Episodic memory deficits were associated with increased tau-PET uptake in the temporal, frontal lobes extensively, and in the supramarginal gyri, the precuneus and posterior cingulate cortex. Associations between semantic memory impairment and tau-PET uptake were much less widespread and involved anterior temporal regions only, on the left hemisphere more than the right hemisphere. Executive dysfunction was related to higher tau-PET uptake in bilateral fronto-parietal regions. The correlation analyses between language and tau-PET uptake showed significant associations in the left greater than right hemispheres, specifically in posterior superior temporal regions, parietal regions involving supramarginal gyrus and angular gyrus, and in inferior and middle frontal gyri. The correlation with praxis revealed significant associations with tau-PET uptake in right greater than left occipital, frontal and parietal regions extensively, and in temporal posterior regions. Adjusting the correlation analyses for global (average across all regions) tau-PET uptake, the core regions of the patterns remained the same (e.g., left temporal regions for semantic memory, left inferior frontal gyrus and temporal regions for language, and right inferior operculum and occipital regions for praxis; Fig. 1, bottom panel). Adding years of education as covariate in the correlation analyses did not change the results (Supplementary Fig. 1), as well as repeating the analyses after excluding young controls from the w-score computation (Supplementary Fig. 2).

**Fig. 2:**
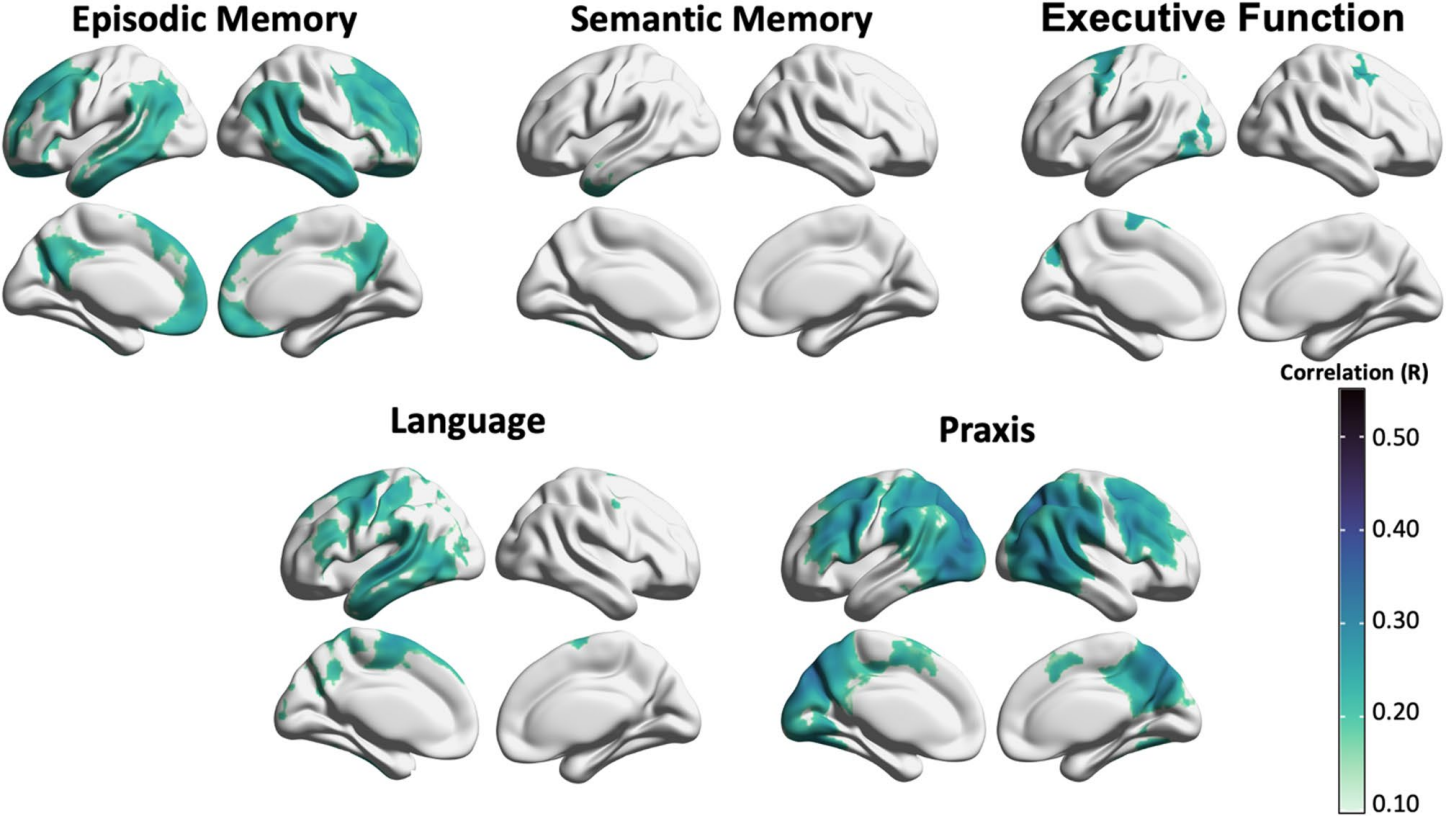
Voxel-wise correlation maps between tau-PET SUVR values and domain-specific cognitive impairment at baseline. The surface map indicates the correlations which survived stringent threshold of pFWE < 0.05

Voxel-wise regression analysis identified similar patterns of tau-PET uptake associated with domain-specific cognitive performance as for the regional correlation analyses (Fig. 2). The core regions of the patterns were the same, however the spatial extent was larger in regional analyses than in voxel-wise results. In particular, with the voxel-wise approach, the executive-related tau-PET pattern was limited to frontal/motor areas, and the left lateralization of the language-related pattern was more accentuated.

### Associations between tau-PET imaging at baseline and domain-specific cognitive decline over time

**Fig. 3:**
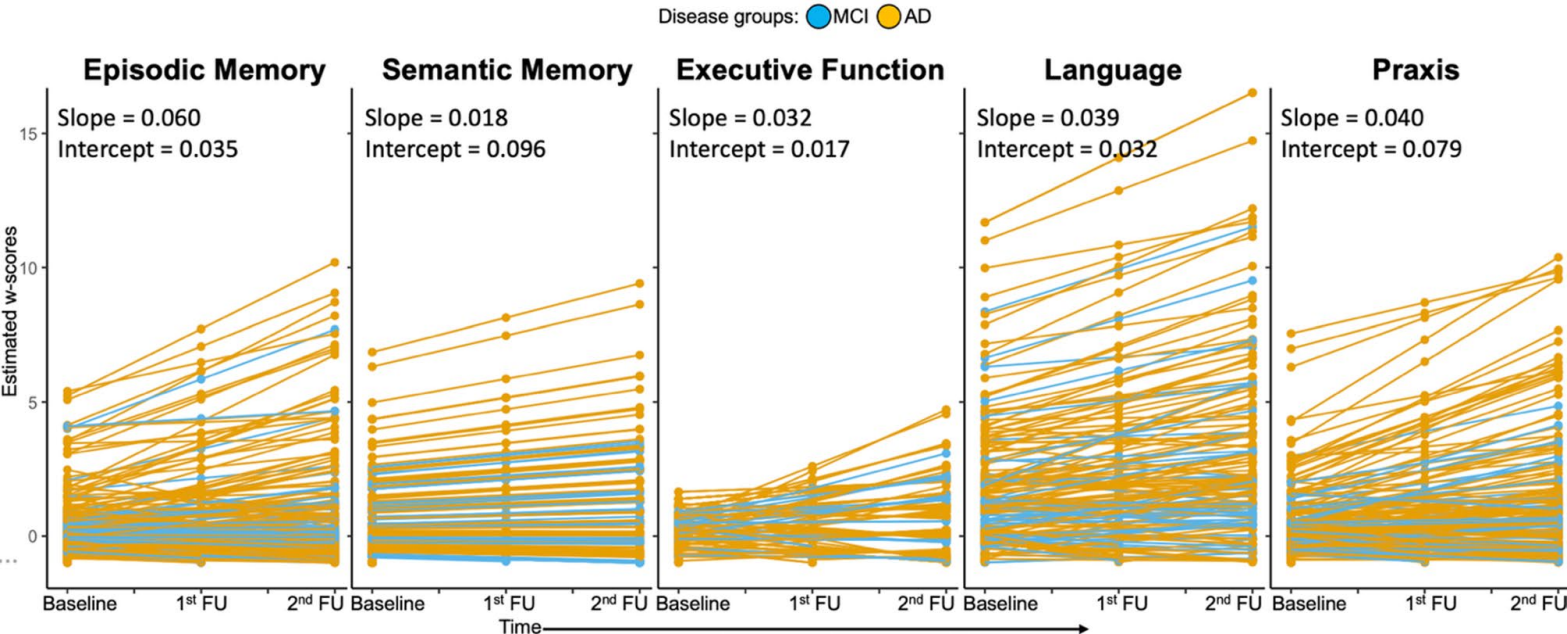
Longitudinal changes in domain-specific composite scores. Individual trajectories are represented using the intercepts (performance at baseline) and slopes (rate of change), estimated with the latent growth curve modelling approach on annualized w-scores. Blue indicates participants with MCI due to AD, and yellow indicates those with mild AD dementia

**Fig. 4:**
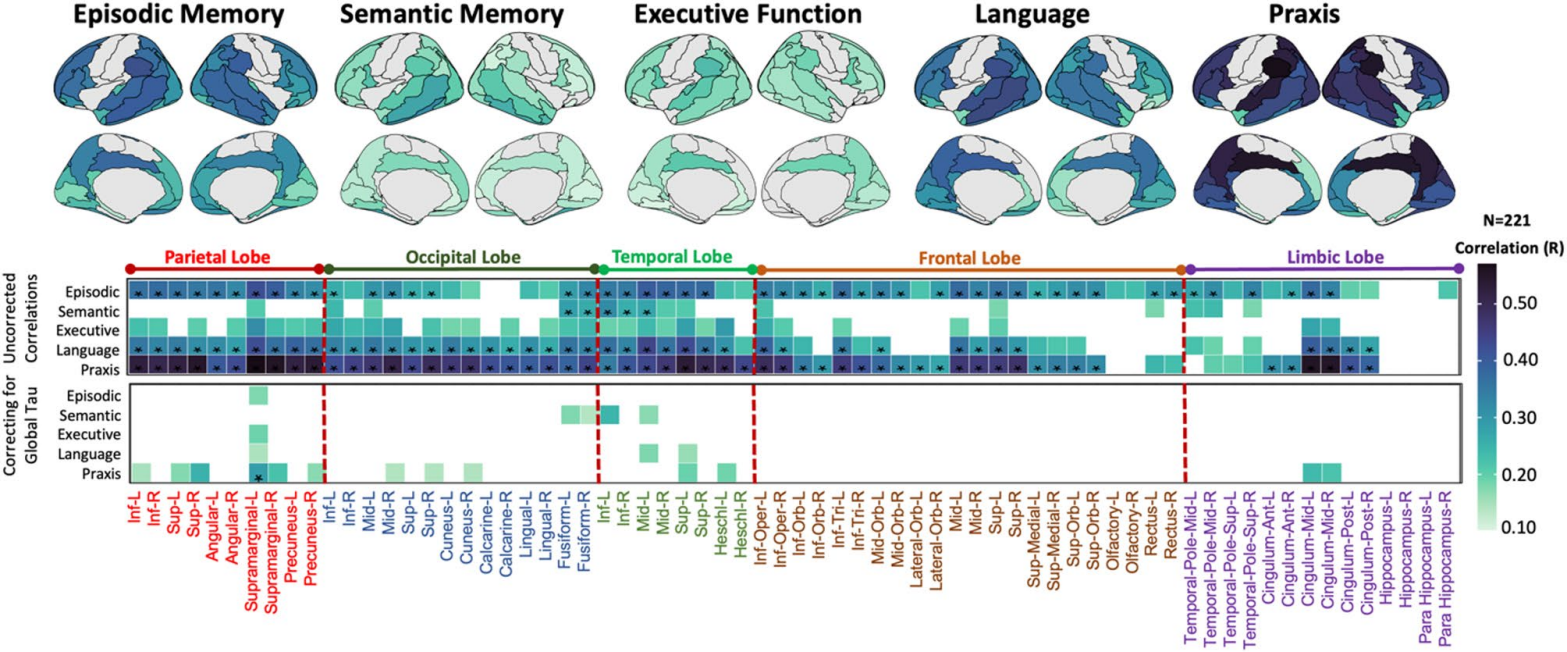
Correlations between regional tau-PET SUVR values at baseline and domain-specific cognitive decline over time (slope). The surface maps display all the regional correlations assessed, while the bottom heatmaps highlights only those correlations which have *p* < 0.05 (with and without correcting for global Tau PET signal). The * indicates the correlations which survived stringent threshold of pFWE < 0.05. The x-axis of heatmap groups AAL brain regions by lobes, and the y-axis represents cognitive domains

**Fig. 5:**
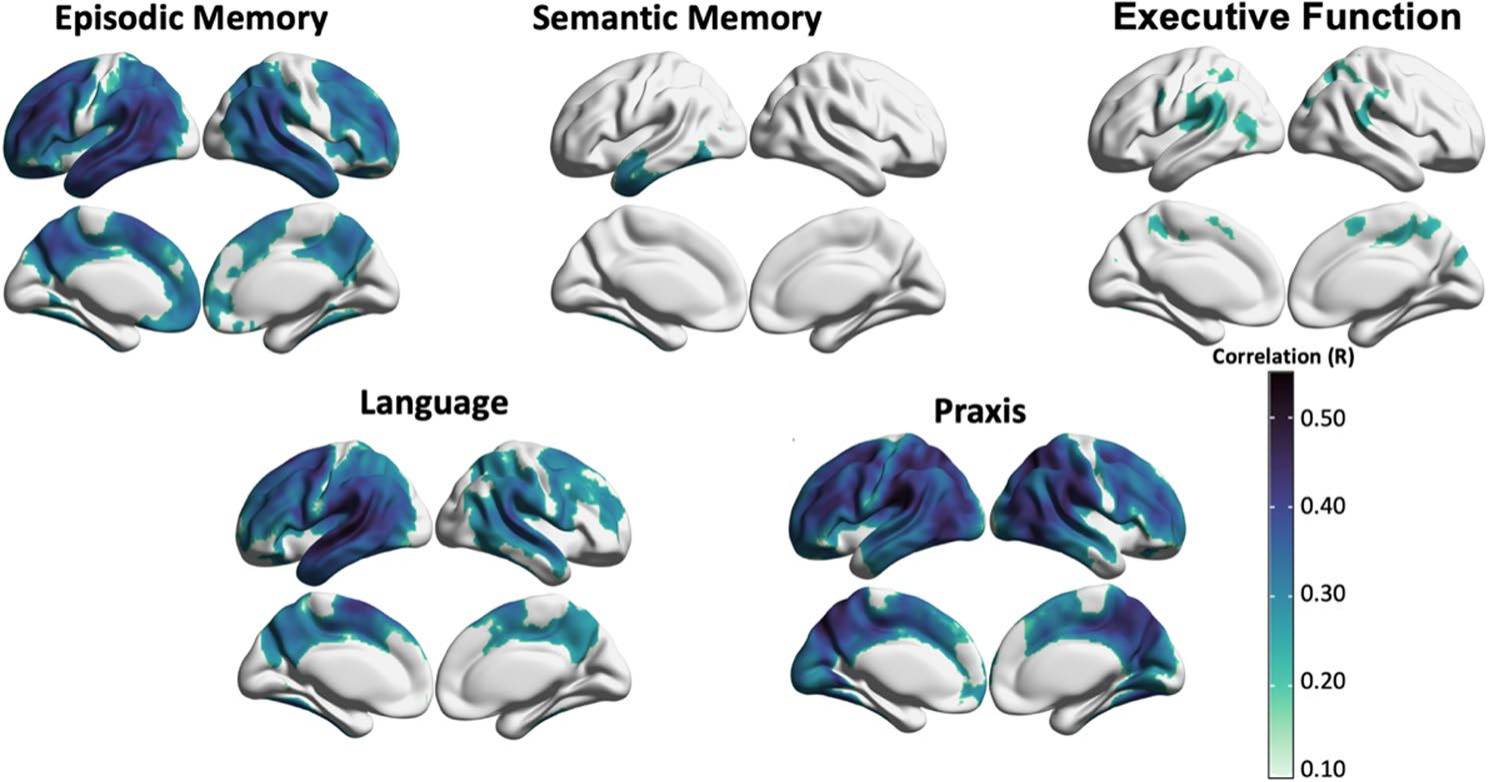
Voxel-wise correlation maps between baseline tau-PET SUVR values and domain-specific cognitive decline over time (slope). The surface map indicates the correlations which survived stringent threshold of pFWE < 0.05

The LGCM of longitudinal cognitive scores fit the data adequately (RMSEA = 0.00, CFI = 1, SRMR = 0.00) for all cognitive domains. Next, we tested for associations between baseline tau-PET and domain-specific cognitive decline over time. Most patients experienced worsening across different domains as shown in Fig. 3 (see Table 2 for summaries across cohorts). The results indicate that there is a global effect of tau, as tau-PET in specific brain regions is associated with cognitive decline across multiple domains. However, differential tau-PET patterns were associated with domain-specific cognitive decline (Fig. 4). Specifically, higher tau-PET uptake in fronto-temporal, parietal, cingulate and limbic regions was indicative of a faster decline in episodic memory. Higher tau-PET uptake in the left anterior and inferior temporal lobe was associated with greater decline in semantic memory. Executive decline exhibited minimal inter-individual variability, possibly due to low dynamic range in this domain (Fig. 3), thus correlations with baseline tau PET did not highlight strong association patterns. Additionally, a more extensive tau-PET uptake pattern in fronto-temporal and parietal regions (L > R), and fronto-parietal and occipital regions was linked to accelerated decline in language and praxis domains, respectively. Adjusting the correlation analyses for global (average across all regions) tau-PET uptake, the domain-specific patterns were more spatially limited and reduced to core regions (e.g., left temporal regions for semantic memory and language, and parietal-occipital regions for praxis; Fig. 4, bottom panel). Voxel-wise regression analysis revealed similar patterns of tau-PET uptake associated with domain-specific cognitive decline (pFWE < 0.05, Fig. 5).

### Associations between Gray matter volume at baseline and domain-specific cognitive decline over time

**Fig. 6:**
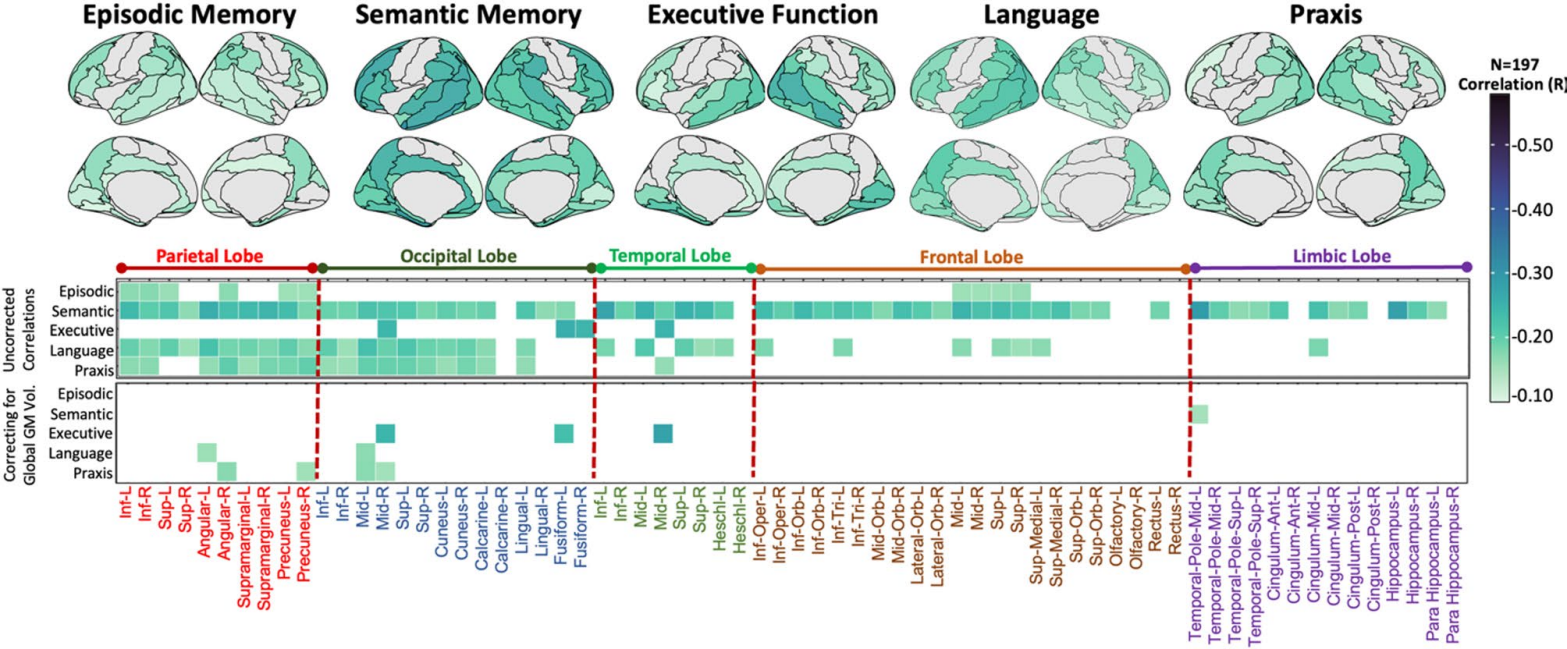
Correlations between regional gray matter volumes at baseline and domain-specific cognitive decline over time (slope). The surface maps display all the regional correlations assessed, while the bottom heatmap highlights only those correlations which have *p* < 0.05 (with and without correcting for global grey-matter volume). The * indicates the correlations which survived stringent threshold of pFWE < 0.05. The x-axis of heatmap groups AAL brain regions by lobes, and the y-axis represents cognitive domains

Next, we examined the associations between baseline atrophy (gray matter volume) and domain-specific cognitive decline over time. The results (Fig. 6) indicate a common pattern of atrophy in temporoparietal regions associated with cognitive decline across all domains. However, unique differential atrophy patterns were also observed, showing associations with domain-specific cognitive decline. Adjusting the correlation analyses for global (sum across all regions) grey-matter volumes, the domain-specific patterns were spatially limited to core regions (e.g., left temporal pole for semantic memory and right parietal-occipital regions for praxis; Fig. 6, bottom panel).

### Association between baseline tau-PET and domain-specific cognitive decline controlling for atrophy

**Fig. 7:**
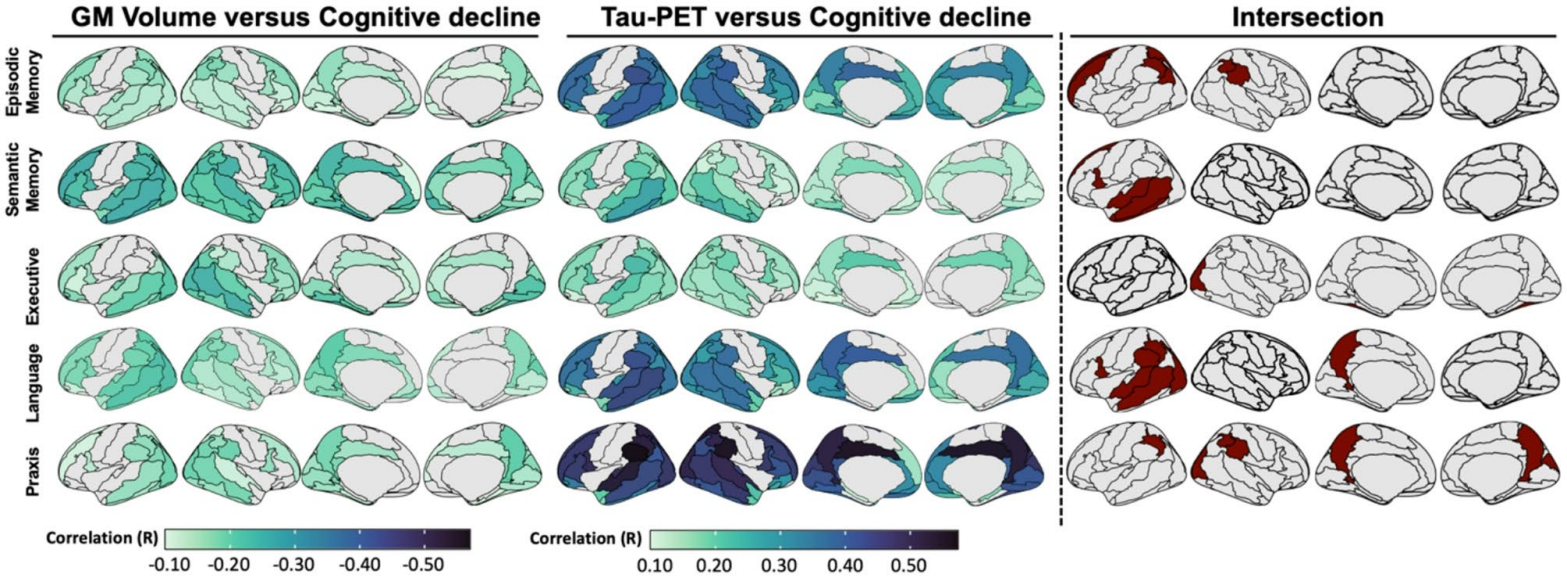
Overlapping regional patterns selected for the mediation analyses. Regions were selected based on the associations of tau-PET and gray matter volumes, respectively, with cognitive decline in each domain. Top-most 10 regions in terms of correlations (*p* < 0.05) were selected for finding the overlap

To further examine whether grey matter atrophy mediates the relationship between baseline tau-PET and longitudinal cognitive decline, we created overlap maps that included regions showing significant association between cognitive performance and both tau-PET uptake and grey matter volume (at *p* < 0.05 uncorrected). The threshold of these analyses was lowered as only a limited number of regions were obtained by associating cognition and grey matter volume. The top 10 regions were assessed from both the categories for assessment of overlap (Fig. 7). The mean tau-PET uptake and grey matter volume were then extracted in the overlap regions and included in mediation analyses, with grey matter volume as the mediator variable (Table 3).

**Table 3:** Summary of the mediation analyses in the brain areas where both flortaucipir tau-PET SUVR and grey matter volume were related to cognitive domain scores (*P* < 0.05)

Cognitive domains	ACME		ADE	
	Estimates	*p*-values	Estimates	*P*-values
Episodic memory	0.0169	0.12	0.7076	< 0.001
Semantic memory	-0.0118	0.34	0.2268	< 0.001
Executive function	0.0300	0.14	0.2717	0.008
Language	0.0142	0.50	0.8191	< 0.001
Praxis	0.0271	0.02*	0.9644	< 0.001

Abbreviations: ACME = average causal mediation effect; ADE = average direct effect

We observed no mediation effect of gray matter atrophy on the association between baseline tau-PET and decline in cognitive domains (Table 3; episodic memory: ACME = 0.02, *P* = 0.12; ADE = 0.71, *P* < 0.001; semantic memory: ACME= -0.01, *P* = 0.34; ADE = 0.23, *P* < 0.001; executive function: ACME = 0.03; ADE = 0.27, *P* < 0.001; language: ACME = 0.01, *P* = 0.50; ADE = 0.82, *P* < 0.001), except praxis domain where marginal mediation effect of gray matter atrophy was observed (praxis: ACME = 0.03, *P* = 0.02; ADE = 0.96, *p* < 0.001).

## Discussion

Our study corroborates the prognostic value of tau-PET regional distribution on cognitive decline in several domains, in a clinical trial cohort patients with MCI-to-mild dementia. Differential tau-PET patterns are associated with domain-specific cognitive performance and decline in symptomatic amyloid-positive patients, as shown by both regional correlation and voxel-wise regression analyses. Specifically, higher tau-PET SUVR in an extensive regional pattern encompassing fronto-temporal and parietal regions was predictive of faster decline in episodic memory. Higher tau-PET uptake in the left anterior temporal lobe related to worse semantic memory, while a more extensive and asymmetric tau-PET uptake pattern in (L > R) fronto-temporal and parietal regions and (R > L) fronto-parietal and occipital regions related to faster decline in language and praxis, respectively. Executive decline showed small inter-individual variability, leading to limited association patterns. Region-based analyses highlighted similar tau-PET uptake patterns as predictive of domain-specific decline.

Previous cross-sectional studies on a similar but smaller cohort showed similar tau-PET uptake patterns associated with cross-sectional domain-specific impairment [8, 9]. Thus, the current study replicates and extends previous evidence. Beyond cross-sectional studies, only a limited number of observational and smaller studies have shown associations between regional patterns of tau-PET and cognitive decline in patients on the spectrum of AD. In 152 individuals with CDR ≤ 0.5, with only a small portion of positive amyloid PET scans, retrospective longitudinal analyses indicated that tau-PET had the largest predictive value on episodic memory and executive functioning decline, as compared to amyloid-PET and structural MRI [21]. Moving from pre-clinical to dementia stages on the AD spectrum, in a small cohort (*n* = 36) of amyloid-positive patients with AD, differential voxel-wise patterns of tau-PET at baseline were found to be strongly associated with subsequent cognitive decline in memory, executive and instrumental functions, while no associations were found with baseline amyloid load and regional cortical atrophy [22]. In a larger cohort of amyloid-positive individuals (*n* = 131) from the ADNI study, widespread associations between Flortaucipir PET patterns and cognitive decline were found in domain-related regions, as compared to limited cortical thickness-cognition associations [23]. Another ADNI-based study on 140 amyloid-positive individuals confirmed the previous results, reporting that cognitive domain-specific tau-PET outperforms amyloid-PET and structural MRI, but also global and temporal lobe tau-PET, for predicting future cognitive decline in episodic memory, language, executive functioning, and visuospatial abilities [24]. Overall, these observational studies indicated that domain-specific cognitive decline in patients with AD reflects underlying tau progression into associated brain circuits, and spatial patterns of tau-PET may have better sensitivity to capture differential decline than MRI-derived measures.

Our study complements and expands on this series of reports. Specifically, our results point to similar tau-cognition spatial relationships in a larger and more diverse trial-based cohort of amyloid-positive patients with a clinical diagnosis of MCI or mild AD dementia. We focused on early stages of the disease as this may be the most efficacious time window to intervene with disease-modifying therapies, and prognostic biomarkers may be the most appropriate tools to stratify patients at these early stages of disease. Notably, our results showed that the observed asymmetry and spatial differences in the prognostic value of tau-PET for cognitive decline are more pronounced than in cross-sectional correlation patterns with cognitive domains. This aligns with recent evidence suggesting that asymmetric tau deposition is associated with earlier disease onset, greater pathological burden, and more rapid cognitive deterioration compared to symmetric patterns [25, 26]. In addition, our results identify overlapping patters of tau-PET in correlation analyses with episodic memory and praxis performance. This overlap is consistent with evidence that tau pathology in regions such as frontal and parietal cortices is associated with decline across multiple cognitive domains, reflecting the interconnected nature of neural networks supporting memory and praxis. However, episodic memory deficits were related to tau-PET in temporal regions more extensively than praxis, while the latter was associated with a tau pattern involving posterior regions, including occipital cortex, that were not part of the episodic memory related patterns (Figs. 2 and 5). These findings highlight the importance of considering not only the overall burden but also differential spatial distributions and the lateralization of tau pathology when assessing prognosis and tailoring interventions in AD.

Across domains, we observed overlapping regions where tau-PET uptake, grey matter volume, and cognition were significantly associated. This convergence, particularly in temporoparietal and medial temporal areas, highlights the close interplay between tau pathology, neurodegeneration, and cognitive decline. Such overlap supports the hypothesis that regional tau accumulation may drive local atrophy, which in turn contributes to domain-specific cognitive deficits [27]. The associations between baseline gray matter volume and domain-specific cognitive decline, while showing some overlap, were less extensive and strong as compared to the associations between baseline tau-PET and domain-specific cognitive decline. This suggests that tau-PET may be a stronger predictor of domain-specific longitudinal cognitive decline than gray matter atrophy; and while structural brain changes are important, they may not capture the full extent of the neurodegenerative processes affecting cognitive functions. This aligns with cross-sectional multimodal imaging studies on the AD spectrum, showing tau-PET uptake in areas without overt neurodegeneration [4, 5, 10, 28]. Tau pathology may locally precede neurodegeneration, as also suggested by significant associations of baseline tau-PET patterns with prospective atrophy as measured by structural MRI [29, 30] and retrospective longitudinal atrophy (years preceding tau-PET) in both cognitively unimpaired individuals and patients with clinical AD [31, 32]. Moreover, our findings suggest that while there are regions where tau-PET signals and gray matter atrophy are both associated with cognitive decline, gray matter atrophy does not mediate the relationship between baseline tau-PET and cognitive decline. This indicates that tau-PET may be a robust and independent predictor of domain-specific cognitive decline. Baseline MRI may also capture more of the normal variation in brain structure, whereas tau-PET uptake is indicative of pathological process. Further research in clinical settings is necessary to confirm these findings and explore potential underlying mechanisms.

Previous studies have described strong associations between baseline tau-PET and cognitive changes over time across the AD clinical spectrum. In head-to-head comparisons, tau-PET binding in temporo-parietal regions outperformed amyloid-PET and structural MRI measures in predicting global cognitive decline [12–14], especially in patients at early AD stages [12]. We relied on ADAS-cog as single tests with domain-specific sub-scores, rather than focusing on extensive cognitive batteries, in order to improve prognosis in clinical practice and trials in a feasible and pragmatic way while capturing different domains. Although a single test cannot fully assess patients’ cognitive functioning and identify co-occurring deficits that a more extensive battery might reveal, it still holds value. Corroborating previous results by transitioning from composite scores to crude single-test sub-scores offers multiple advantages. First, administering a single test is quicker and less resource-intensive, making it suitable for settings with time constraints or limited access to testing materials, such as clinics that will soon start implementing disease modifying treatment delivery. Second, shorter testing time minimizes fatigue and stress for patients, which can result in more accurate and reliable performance. Third, a single-test assessment is easier to repeat over time for monitoring changes in cognitive function, such as in longitudinal studies or ongoing treatment assessments, reducing data missingness and subjective variability in multiple-test battery administration.

As compared to previous reports, we also implemented a different approach to quantify cognitive changes over time, utilizing LGCM. This analytic approach offers several advantages over other previously used approaches, such as linear mixed effects models and the analysis of absolute differences for tracking changes over time, and has been successfully implemented in prognostic analysis in dementia [14, 33–35]. In particular, LGCM (i) explicitly models individual differences in initial status (intercept) and rate of change (slope), allowing for a detailed understanding of how individuals differ in their growth trajectories; (ii) handles missing data more robustly through full information maximum likelihood estimation, which uses all available data points and provides unbiased parameter estimates under the assumption that data are missing at random; (iii) offers a range of fit indices to assess model fit and compare alternative models, facilitating the identification of the best-fitting growth model over complex growth models [36]. LGCM may be a powerful tool to implement in future AD clinical trials, as it provides a more nuanced, flexible, and robust approach to tracking changes over time compared to linear mixed effects models and the analysis of absolute differences.

Notably, for both cross-sectional and longitudinal analyses in our cohort, after adjusting for global tau-PET uptake and global grey-matter volume, the observed domain-specific associations were spatially limited to core brain regions for each cognitive function. This spatial limitation highlights the importance of regional pathology over global disease burden in driving specific cognitive deficits, and the selective vulnerability of functionally specialized brain networks. Regional localized patterns of tau accumulation predict domain-specific cognitive decline and may inform both the timing and type of interventions. These findings reinforce the value of regional PET assessment and support the use of targeted approaches in both research and clinical interventions. Early detection of regionally restricted tau could enable targeted therapies and tailored clinical management, supporting a personalized medicine approach to delay or mitigate decline in specific cognitive domains.

Our study has some limitations. First, the number of cancellation tests (part of ADAS-Cog13) was not available for participants in the A05 study, limiting our evaluation of executive domain to the AMARANTH and EXPEDITION studies. and we acknowledge that ADAS-Cog13 is able to evaluate more sensitively some cognitive domains (i.e., memory) than others. Additionally, w-scores for the executive domain were not calculated as controls had ADAS-Cog11 scores only available. Second, the control group was not age-matched, as it included participants across a wide age range. Notably, some control participants were younger than 50 years old (*n* = 15, mean age = 29.0), which may introduce some biases in the representativeness of the cohort. The inclusion of younger individuals could lead to an overestimation of cognitive differences between patients and controls, as younger participants are likely to perform better on cognitive assessments due to age-related advantages in processing speed and memory. Third, our mediation analyses were restricted to evaluating local relationships and did not account for potential distant relationships between tau-PET, gray matter atrophy, and domain-specific cognitive performance/decline. Finally, participants were recruited from expert clinical trial centers under highly controlled conditions, introducing selection bias. This might not accurately represent the broader patient population actually seen in the clinics. Additionally, the study population lacked racial and ethnic diversity, with over 90% of participants being white. The association between tau, neurodegeneration and cognition may vary based on race and ethnicity as proxies for social determinants of health [37]. Other individual factors can also modify the relationship between tau accumulation and cognition, with younger age, female sex, higher education and cortical thickness being related to increased reserve and resistance against the effect of pathology on cognitive performance [38]. Much more work in diverse cohorts is needed to validate our results in population-representative cohorts.

In summary, we showed that differential regional patterns of tau-PET are associated with domain-specific cognitive decline in a large cohort of patients with MCI and early AD dementia. Implementing latent growth curve modelling approaches on single-test longitudinal changes may empower prognosis and outcome prediction. Tau-PET may be a good tool to stratify patients in clinical trials of disease-modifying therapies, with personalized predictions of neurodegeneration and cognitive trajectories, enhancing the chance to identify the best time window and cohort where a given therapy can be most effective.

## Supplementary Information

Below is the link to the electronic supplementary material.

Supplementary Material 1

## Data Availability

The datasets used and/or analysed during the current study are available from the corresponding author on reasonable request. For the purpose of open access, the authors have applied a CC BY public copyright licence to any Author Accepted Manuscript version arising from this submission.
