# Supplementary material for: Regional tau PET patterns predict prospective domain-specific cognitive decline in early symptomatic Alzheimer’s disease
Source: Alzheimers Res Ther. 2025 Oct 6;17:220. doi: 10.1186/s13195-025-01868-7 (PMC12502172; doi:10.1186/s13195-025-01868-7)
Supplement: Supplementary file 1 — Supplementary Material 1 [file 13195_2025_1868_MOESM1_ESM.docx]

**Supplementary Material**

**Regional tau PET patterns predict prospective domain-specific cognitive decline in early symptomatic Alzheimer’s disease**

Maura Malpetti^1,2*^, Saima Rathore^3*^, Leonardo Iaccarino^3^, Renaud La Joie^4^, Giulia Tronchin^3^, Alette M. Wessels^3^, John R. Sims^3^, Michael J. Pontecorvo^3^, Sergey Shcherbinin^3^, Gil D. Rabinovici^4,5^

1 Department of Clinical Neurosciences and Cambridge University Hospitals NHS Trust, University of Cambridge, Cambridge, UK

2 UK Dementia Research Institute at University of Cambridge, Cambridge CB2 0XY, UK

3 Eli Lilly and Company, Indianapolis, USA

4 Memory and Aging Center, Department of Neurology, Department of Neurology, Weill Institute for Neurosciences, University of California, San Francisco, San Francisco, California, USA

5 Department of Radiology and Biomedical Imaging, University of California, San Francisco, San Francisco, California, USA

*equal contribution

***Corresponding author:***

*Dr. Maura Malpetti*

*Email: mm2243@medschl.cam.ac.uk*

*Department of Clinical Neurosciences*

*University of Cambridge*

*Herchel Smith Building, Forvie Site*

*Robinson Way, Cambridge Biomedical Campus*

*Cambridge CB2 0SZ*

**Supplementary Figure 1:** **Correlations between regional tau-PET SUVR values and domain-specific cognitive impairment at baseline with and without correcting for years of education.** The surface maps display all the regional correlations assessed, while the bottom heatmap highlights only those correlations which have p<0.05. The * indicates the correlations which survived stringent threshold of pFWE<0.05. The x-axis of heatmap groups AAL brain regions by lobes, and the y-axis represents cognitive domains.

*
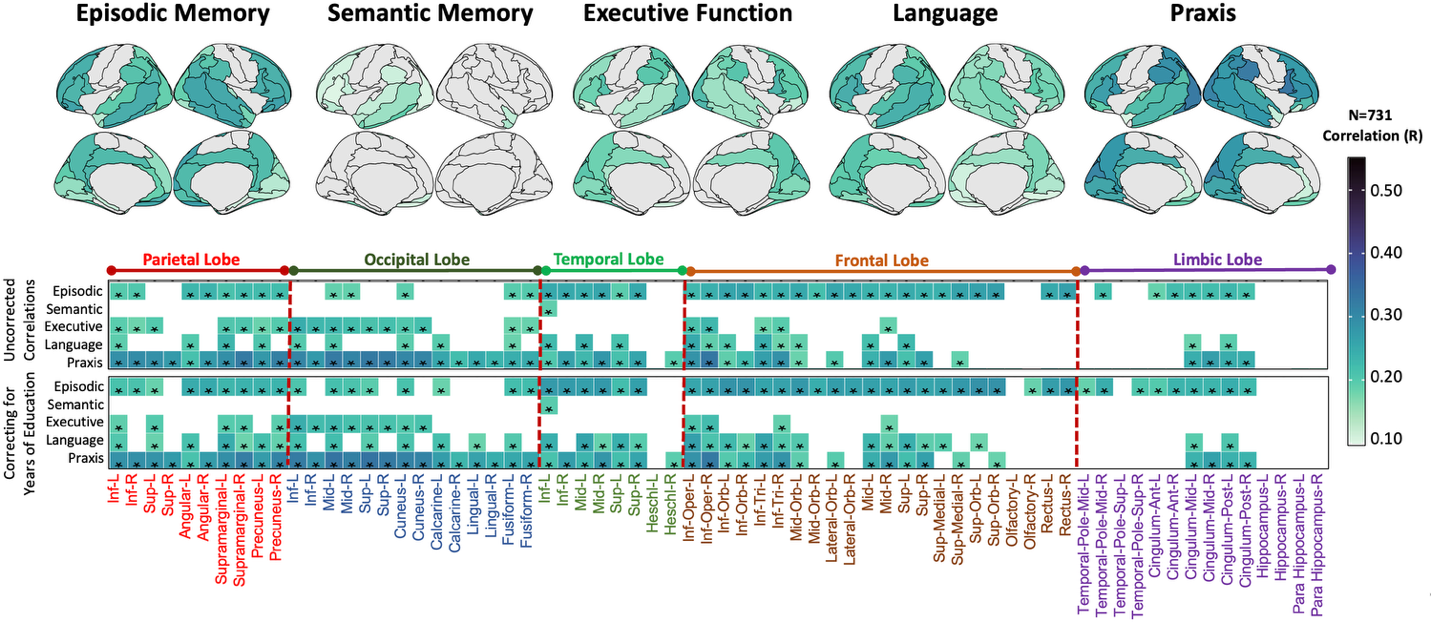
*

**Supplementary Figure 2:** **Correlations between regional tau-PET SUVR values and domain-specific cognitive impairment at baseline with and without leveraging younger controls for calculation of w-scores.** The surface maps display all the regional correlations assessed, while the bottom heatmap highlights only those correlations which have p<0.05. The * indicates the correlations which survived stringent threshold of pFWE<0.05. The x-axis of heatmap groups AAL brain regions by lobes, and the y-axis represents cognitive domains.

*
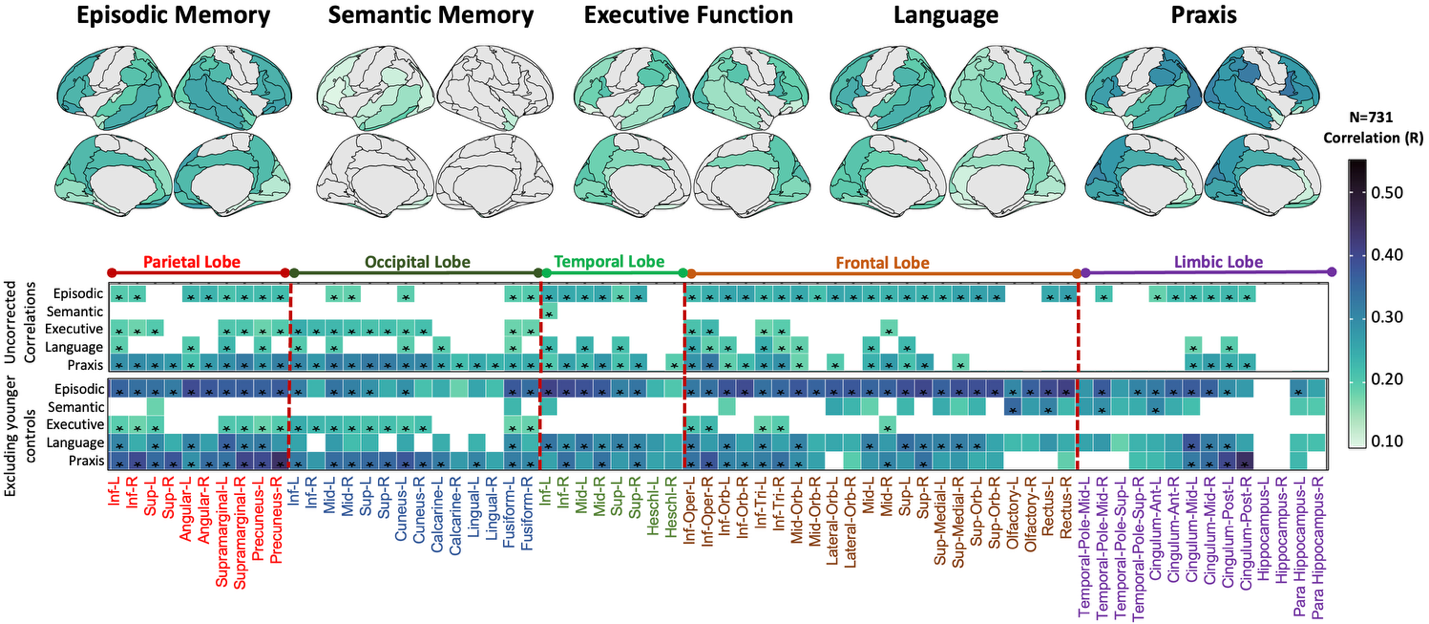
*
